# A review on recent advances in Alzheimer's disease: The role of synaptic plasticity

**DOI:** 10.3934/Neuroscience.2025006

**Published:** 2025-04-15

**Authors:** Nour Kenaan, Zuheir Alshehabi

**Affiliations:** 1 Faculty of Medicine, Tishreen University, Latakia, Syrian Arab Republic; 2 Department of Pathology, Tishreen University Hospital, Latakia, Syrian Arab Republic

**Keywords:** neuroplasticity, Alzheimer's disease (AD), treatments, genetic mutations, sex hormones, gut-brain axis

## Abstract

Alzheimer's Disease (AD) remains a significant global health challenge, characterized by progressive neurodegeneration and a decline in cognitive abilities such as memory and learning. Despite being the main cause of dementia worldwide, the precise mechanisms that underlie neuronal dysfunction and synaptic plasticity impairment in AD remain elusive. However, while genetic mutations, dietary factors, and immune dysregulation are implicated in AD pathogenesis, the current therapeutic approaches are largely centered around acetylcholinesterase inhibitors (AChEIs). Nevertheless, this cholinergic hypothesis of AD is no longer satisfactory in describing this disease and has demonstrated a limited efficacy. Hence, new treatment approaches should be developed, and that requires us to view AD from a new perspective. Herein, in our review, we present the latest studies that discussed possible AD pathologies and pharmacotherapies. Additionally, we highlight that the emerging treatments that precisely targets brain regions associated with enhancing neuroplasticity have delivered promising results and seem to be more effective than older treatments. Finally, by viewing AD as a complex interplay of various factors that ultimately cause synaptic dysfunction and cognitive decline, we can develop more effective therapeutic interventions and ultimately alleviate the significant burden of this debilitating disease for both patients and their families.

## 1. Introduction

According to The World Health Organization's (WHO) 2022 statistics, 55 million people worldwide suffer with dementia. It is estimated that every year there are nearly 10 million new cases of dementia, with Alzheimer's causing 60%–70% of them. In addition to dementia being the seventh leading cause of death, it imposes an enormous economic burden due to the dependency and disability that are caused among elderly patients [1].

Alzheimer's disease (AD) is a degenerative brain disorder that slowly diminishes the patients' life quality, thus causing the loss of significant cognitive functions of the brain including thinking and memory storage [2]. AD is often identified by genetic mutations in the Amyloid precursor protein (APP), presenilin 1 (PSEN1), or presenilin 2 (PSEN2). Commonly, when AD is diagnosed <65 years of age, it is referred to as early-onset (EOAD), accounts for less than 5% of the cases, and is usually related to one of the severe genetic mutations. Late-onset (LOAD: >65 years of age) is the most common form of AD, accounts for over 95% of AD cases, and is most commonly related to the APOE-epsilon 4 polymorphism [3].

However, the specific mechanism responsible for causing AD is still poorly understood, and several theories have tried to define that more clearly. Recent findings have suggested the role of genetic mutations in AD epidemiology, such as mutations in the processing of Amyloid beta (Aβ) peptide and Tau protein, dysfunctions of synaptic and mitochondrial proteins, neurovascular alterations, oxidative stress, and neuroinflammation, hence the disruption of brain plasticity [3].

Moreover, studies have demonstrated that AD patients have an altered gut microbiome (GMB), which can alter microbial-derived metabolites and peripheral immunity, and could potentially alter the central nervous system (CNS immune response in the context of neurological diseases [4]. Another interesting suggestion would be the role of sex hormones as risk factors in AD, as it is estimated that the lifetime risk for dementia in women at the age of 45 is 20% compared to 10% of men at the same age [5].

Neuroplasticity (also known as neural plasticity) is an important concept to mention. This represents the brain's ability to adapt to intrinsic or extrinsic stimuli. This adaption process includes modifications in the molecular environment of the neural structure and can involve functional changes in response to brain damage or structural changes due to learning. In recent years, many published studies have explained how neuroplasticity weakens in diseases and illnesses that affect the brain's structure [6].

In this review, we demonstrate the recent findings and research that elaborate on these suggested concepts for having a defined understanding of AD and its treatments, including the latest suggested methods for enhancing intellectual and mental activities in Alzheimer's patients.

## 2. Discussion

### 2.1. Genetic mutations in AD epidemiology

The role of genetic mutations in degenerative diseases is a common concept. By considering AD as a neurodegenerative disease, several studies have demonstrated important mutations that play a critical part in AD occurrence, more specifically, mutations that led to the aggressive production of the β-amyloid (Aβ) peptide.

A systematic review was performed to explore the spectrum of variants in genes that are linked with AD (APP, PSEN1, and PSEN2 genes) among 22 Arab countries. The researchers systematically studied 18 studies including a total of 2173 individuals, among which 888 were clinically diagnosed with AD. The results revealed that 27 variants in 8 genes were linked to AD. Surprisingly, 17 of these variants were particularly found in the Arab population, whereas 10 variants were shared with other ethnic groups [7].

A systematic review was recently published that investigated the risk factors behind AD, including significant mutations such as the Swedish mutation (K>M670/671N>L) and the A673>V mutation. This study discussed how the Aβ peptide is cleaved by the α-secretase, β-secretase, and γ-secretase enzymes close to the pathogenic APP and the mutations chromosomal sites. The researchers concluded that the pathogenic mutations (Swedish mutation (M670>K/N671>L) and A673>V mutation) were present at the exact position of the APP. Nevertheless, the A673>T mutation has revealed protection against AD [8].

Another study was performed to analyze the association between neurodegenerative disorders (including AD) and the chromosome 9 open reading frame 72 (C9orf72) GGGGCC (G4C2). The study included 27 case-control studies, thereby incorporating 7202 AD patients along with other ND cases. The results showed that the C9orf72 repeat expansions (>30) were associated with AD (OR = 4.88, 95% CI = 2.71–8.78). However, the intermediate repeat expansions of C9orf72 (20–30) were not associated with AD (AD: OR = 1.16, 95% CI = 0.39–3.45;). The researchers suggested that measuring the C9orf72 G4C2 repeat expansions may be helpful in the early-stage differential diagnosis of different NDs [9].

Another interesting meta-analysis indicated that clonal hematopoiesis of indeterminate potential (CHIP)-associated mutations could alter AD progression. Since CHIP-associated mutations are known to alter the development and function of myeloid cells, these mutations may have a major role in AD [10]. To investigate this, the researchers analyzed blood DNA sequencing data from 1362 individuals with AD and 4368 individuals without AD. They found that the patients with CHIP had a lower risk of AD [odds ratio (OR)  =  0.64, P =  3.8  ×  10^−5^], alongside Mendelian randomization analyses that supported the association. Moreover, in seven of eight CHIP carriers, the same mutations in the microglia-enriched fraction of the brain were noticed. In six CHIP carriers, the results indicated a large proportion of the microglial pool in the mutated cells from the samples examined [10].

Several studies have demonstrated the role of apolipoprotein as a genetic risk factor for AD. One important apolipoprotein gene that is considered the highest genetic risk of AD is APOE4. About 25% of people carry one copy of APOE4, and 2 to 3% carry two copies. APOE4 helps regulate cholesterol and unsaturated fatty acid chains in the bloodstream [11],[12].

One particular systematic review included 20 eligible studies that analyzed the association between the APOE ɛ4 allele and the risk of developing AD in global Hispanic populations. The results found that APOE ɛ4 significantly increased the AD risk in this ethnic group (OR = 3.80, 95% CI: 2.38–6.07). However, these data are limited, thus requiring more studies, and the analyses need to be adjusted according to different regions [13].

From another perspective, a review highlighted that persistent APOE4 homozygosity could help cope with heavy enteric infections and malnutrition, specifically in the first two years of brain development. However, under changes in lifestyle and unhealthy diets during ageing, this genotype could lead to severe cognitive impairments and an increased risk of AD [12],[14].

There is no doubt that genetic mutations have a primary role in neurodegenerative diseases. Yet, there remains a lack of precise understanding of how these mutations contribute to AD pathogenesis and how they interact to produce effects at various cellular and biological levels.

### 2.2. Gut-brain axis and neuroinflammation

**Figure 1.**
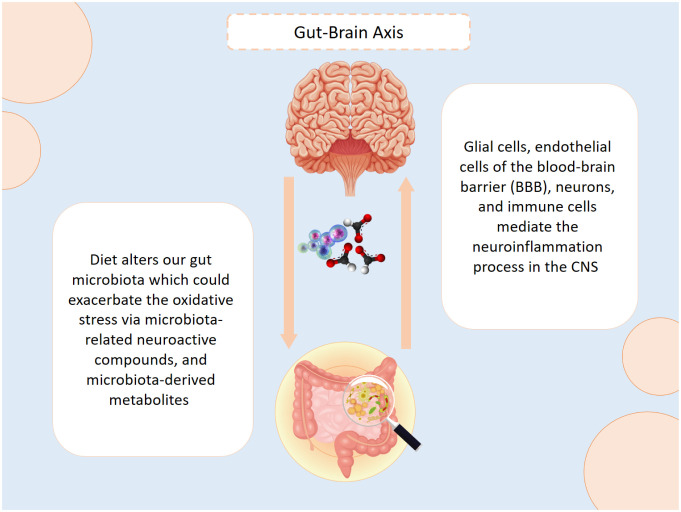
The special connection between the gut and the brain is a way to exchange messages and signals between the two organisms and contribute to the regulation of the internal environment. This gut-brain axis is mainly controlled via the sympathetic and parasympathetic nervous systems, circulatory hormones, and other neuromodulatory agents. Hence, the gut microbiota is involved in the process [Figure was designed by Dr. Kenaan via PowerPoint].

Neuroinflammation is an innate immune response of the CNS initiated by traumatic brain injuries and chronic neurodegenerative diseases. This process is mediated by glial cells, endothelial cells of the blood-brain barrier (BBB), neurons, and immune cells. When the microglia and astrocytes are activated by Amyloid-β (Aβ) plaques they intensely release pro-inflammatory chemokines and cytokines, thus leading to synaptic damage [15]. The association between diet and oxidative stress has gradually gained researchers' interest. Since diet has a significant impact on the gut microbiota, several studies have demonstrated that changes in diet (and consequently changes in gut microbiota) can impact the neuroimmune system, thus initiating or even exacerbating the severity of numerous neurodegenerative diseases including AD. This pathway between the gut and CNS, which is called the gut-brain axis, is bidirectional, and is connected via the circulatory system, immune system, vagus nerve, neuroendocrine system, microbiota-related neuroactive compounds, and microbiota-derived metabolites. Moreover, this pathway is regulated by the sympathetic and parasympathetic nervous systems, circulatory hormones, and other neuromodulatory agents [16] (Figure 1). A systematic review published in 2022 studied the link between the gut microbiota and AD. The researchers included studies published in the last 5 years, in which eight observational studies were selected and performed in humans. The findings suggested that AD patients exhibit a decrease in the richness of the gut microbiota. However, researchers note the need for more human studies, particularly clinical trials, to establish clinical recommendations on this topic. Furthermore, they suggested conducting additional research on the modulating intestinal microbiota [17].

A recently published systematic review aimed to study the impact of Polyphenols on neurodegenerative diseases. Polyphenols are plant-derived compounds produced in response to external stress factors and are found in grapes, blueberries, tea, cocoa, and walnuts [16]. In this study, the researchers included 146 studies, containing 52 animal research and 32 clinical trials. The results from eight included studies on AD patients indicated a higher abundance of Lactobacillus, Streptococcus, Bifidobacterium, Blautia, and Escherichia, and a lower abundance of Alistipes, Bacteroides, Parabacteroides, Sutterella, and Paraprevotella compared to healthy individuals. Additionally, AD patients have exhibited higher levels of pro-inflammatory cytokines such as NLRP3, CXCL2, IL-6, and IL-1β in plasma samples, along with the activation of asparagine endopeptidase (AEP), which is associated with neuroinflammation. Furthermore, they highlighted that polyphenol consumption could inhibit pathogenic bacteria and reduce the pro-inflammatory immune response in the gut, thereby decreasing neuroinflammation [16].

Another research systematically reviewed 30 studies to explain the implications of gut and oral microbiota in neuroinflammation. The researchers demonstrated that the microbiota of AD patients were altered and distinct from those of normal individuals. Furthermore, they clarified that these changes in the gut microbiota were associated with increased polyunsaturated fatty acids metabolites, oxidating enzymes, intestinal permeability, IL-1B, and IL-6 in the plasma. Moreover, they highlighted the role of NLRP3 in neuroinflammation, as its higher levels were correlated with intestinal inflammation and higher levels of blood inflammatory factors. However, the researchers emphasized that a healthy, balanced microbiome positively influences AD treatment, though the exact mechanism underlying this connection remains unclear [18].

Another systematic review investigated whether dietary flavonoids (a subclass of polyphenols) affect the gut microbes and their metabolites. Flavonoids can be found in leafy vegetables, onions, apples, berries, cherries, soybeans, and citrus fruits. The researchers in this study suggested that flavonoids could improve cognitive functions by being absorbed through the intestine, crossing the blood-brain barrier, and entering the brain tissue. Flavonoids deter the expression and secretion of inflammatory factors, hence decreasing the damage caused by oxidative stress. These results demonstrated the potential of diet as both a treatment and preventive approach for neurodegenerative diseases [19],[20].

In recent years, various natural products have garnered attention for their potential benefits in neurodegenerative diseases, particularly AD. Matcha green tea has shown promise in improving cognitive functions and sleep quality in older adults with cognitive decline over a 12-month randomized controlled study [21]. Similarly, chewing Olibanum gum demonstrated clinical efficacy in patients with mild-to-moderate AD in a randomized controlled trial, thus suggesting its potential as an adjunct therapy [22]. Furthermore, citrus supplementation has been explored for its impact on subjective cognitive decline, with a 36-week placebo-controlled trial showing favorable results [23]. These findings align with the growing body of evidence that highlighted the role of phytochemicals in mitigating neurodegenerative conditions. A broader review of natural products targeting protein aggregates offers promising insights into their therapeutic potential for neurodegenerative diseases [24]. The phytochemicals found in various plants, including those in Oroxylum indicum, have also been recognized for their potential in AD treatment [25], further solidifying the importance of exploring plant-based compounds in neurodegenerative disease management.

The emerging evidence suggests that sauna bathing may play a protective role against neurodegenerative diseases such as AD. Regular sauna use has been associated with improved cardiovascular function, reduced inflammation, and enhanced neurotrophic support, all of which are critical factors in brain health. A longitudinal study by Laukkanen et al. (2017) found that frequent sauna bathing was inversely correlated with the risk of dementia and AD in middle-aged Finnish men, thus suggesting a potential neuroprotective effect [26]. Moreover, heat therapy has been investigated as a possible treatment for neurodegenerative diseases due to its ability to reduce oxidative stress and promote the cellular repair mechanisms [27]. The physiological effects of sauna bathing, including increased circulation and heat shock protein activation, have been proposed as mechanisms that could mitigate neuroinflammation, which is a key contributor to AD's pathology [28]. While further research is needed, these findings suggest that sauna therapy could serve as a complementary approach to promote cognitive health and potentially delaying the onset of AD.

### 2.3. Sex hormones association with AD

Approximately two-thirds of AD patients are women. Women face a higher lifetime risk of developing the disease, with a likelihood of 1 in 5 compared to men, who have a risk of 1 in 10. Although it is known that women have a longer lifespan than men, these numbers led scientists to the possibility that sex hormones may play a role in the pathological mechanism of AD [29].

A Mendelian randomization study was conducted to investigate whether genetically predicted sex and growth hormones are correlated to the risk of AD. In this study, the researchers obtained genetic variants from large, published genome-wide association studies (GWAS) and applied them to GWAS of AD based on clinical diagnosis. Additionally, they obtained results using inverse variance weighting with a sensitivity analysis (i.e., MR-Egger, weighted median, and MR-PRESSO), and performed multivariable analyses adjusted for pleiotropic effects and potential sources of selection bias. The researchers found that the risk of paternal AD (having a parent had AD) decreased when the total testosterone levels were higher [odds ratio (OR) 0.86, 95% confidence interval (CI) 0.76 to 0.97, per SD increase in testosterone]. They also concluded that further studies could be done on the association between testosterone and the immune system [30].

Another interesting meta-analysis used a random effect model and included seven prospective cohort studies with a total of 5251 elderly men and 240 cases of AD. The researchers pointed out that lower testosterone levels in elderly men were associated with cognitive declined (random RR = 1.48, 95% CI 1.12–1.96, P = 0.006) [31].

Interestingly, a systematic review was published in 2023 that evaluated studies on the use of hormone replacement therapy (HRT) as a preventive or therapeutic option for AD in women. The researchers included several observational and clinical studies from databases, one of which was the well-known Women's Health Initiative Memory Study (WHIMS). Among the included studies, nine randomized controlled trials (RCTs) had mixed results, five of them showed no substantial differences in cognition or function, while the remaining four indicated progress in cognition. Conversely, observational studies revealed that HRT improves cognitive function across a wide range of neurological and neuropsychiatric tests. Based on all the findings, the researchers concluded that estrogenic intervention has a positive impact on cognitive function when used before neurological insults, particularly in younger women [32].

Based on the discussed evidence, sex hormones are indeed associated with AD progression. However, the mechanism by which hormones can influence this progression remains poorly understood. Therefore, further investigations are needed.

### 2.4. Neuroplasticity in AD patients' brains

Neuroplasticity, or neural plasticity, refers to the brain's capacity to adjust to internal or external stimuli. This adaptation process involves changes in the molecular environment of neural structures and can lead to functional modifications due to brain injury or structural alterations resulting from learning. In recent years, numerous studies have described how neuroplasticity declines in disorders that impact brain structure.

**Figure 2.**
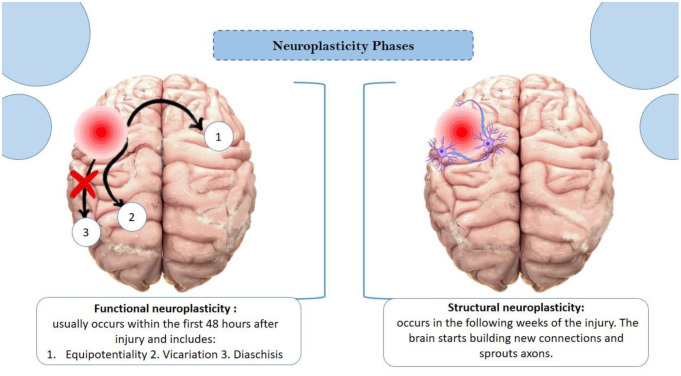
There are two phases of neuroplasticity: 1. Functional neuroplasticity that occurs within 48 hours after injury. This includes three concepts as shown in the figure: a- Equipotentiality (When there is damage to a specific area of the brain, the opposite side of the brain is capable of supporting the lost function); b- vicariation (The brain has the ability to reorganize other areas of the brain in order to take on functions that were not originally their intended purpose); and c- Diaschisis (One region of the brain being damaged could result in the loss of function in another area because of interconnected pathways). 2. On the other hand, structural neuroplasticity occurs in the following weeks, and here the brain starts remodeling itself via synaptic plasticity and axonal sprouting. Structural neuroplasticity could continue for weeks or even months depending on the type of injury [Figure was designed by Dr. Kenaan via PowerPoint].

Functional neuroplasticity refers to the brain's ability to move functions from a damaged brain area to other normal areas. This process mainly includes concepts like equipotentiality, vicariation, and diaschisis [6]. Functional neuroplasticity typically occurs within the first 48 hours after injury, during which cell damage and the loss of certain cortical pathways take place. In contrast, structural neuroplasticity occurs in the weeks following the injury as the brain recruits support cells, builds new connections, and sprouts axons to facilitate remodeling and reorganization around the damaged area [6] (Figure 2).

Several studies have been conducted in recent years to investigate neuroplasticity in the brains of AD patients. A systematic review and meta-analysis were conducted to evaluate the effectiveness of Transcranial Magnetic Stimulation (TMS)-derived measures to distinguish AD and mild cognitive impairment (MCI) from cognitively normal older adults (CN). This review included 163 MCI patients, 1454 AD patients, and 1111 CN participants (totaling 2728 participants). The AD patients exhibited a higher cortical excitability and an impaired cortical plasticity compared to the CN cohorts. Furthermore, this cortical pattern was significantly associated with a lower cognitive performance in AD patients. This study implied the possibility of measuring indicators of cortical activity and plasticity to diagnose and treat AD [33].

Neuroplasticity is closely linked to synapse destruction. A systematic review demonstrated the critical role of astrocytes in synapse formation and elimination in the brains of AD patients. Astrocytes affect the synapse numbers by expressing specific signaling molecules: MERTK, MEGF10, C3, and ApoE4. In addition, damaged glutamate transport and signaling, impaired Ca^2+^ signaling, and decreased TSP1 expression all indirectly decrease the synapse numbers. The researchers systematically analyzed 52 papers published online up to February 2, 2022. The results showed that a reactive phenotype with an altered morphology and gene expression in AD pathology can lead to astrocyte changes, thus resulting in synapse loss and, consequently, the manifestation of disease symptoms. However, potential therapeutic approaches should not necessarily focus on removing the astrocytes, but rather on targeting damaged ones or preventing AD-induced changes in them [34].

In another meta-analysis, the researchers indicated that mitochondrial miRNAs play an essential role in synapse growth [35]. Moreover, they identified several factors that influence mitochondrial function, such as biogenesis, calcium signaling, biological sex, and ageing. It is well known that mitochondria are responsible for generating energy by producing ATP molecules. Furthermore, the brain has the highest energy demand for sustaining ion gradients across cell membranes and for neurotransmission, which requires the availability of several ions. The researchers identified a list of mitochondrial miRNAs that are differentially in AD. Since the neocortex contains approximately 150 trillion synapses responsible for neurotransmission and neurofunction, it is well understood that synaptic dysfunction, and consequently reduced neuroplasticity, could be the major contributor of AD symptoms progression and a potential target for future treatment. However, further research is needed to understand the specific effects of mitochondrial miRNAs on synapse function [35].

Based on the evidence presented, AD can be deduced to be a multifactorial disease in which accumulating oxidative stress, mitochondrial dysfunction, synaptic and neuroplasticity decline, dysfunctional microglia, and genetic mutations all contribute to neurodegeneration [36]. This perspective may prompt a reconsideration of the currently adopted therapeutic approaches for AD.

### 2.5. Current guidelines for AD treatment

Since 2015, acetylcholinesterase inhibitors (AChEIs) medications have been the mainstay of AD treatment based on the “Cholinergic Hypothesis” of AD. However, in 1985, Ashford and Jarvik were the first to suggest that AD affects all levels of neuroplasticity in the brain, which could explain why AChEIs do not halt the disease but only alleviate its symptoms [37].

To date, no definitive treatment for AD has been established. However, guidelines recommend several drug classes that can temporarily improve some symptoms, including the following: acetylcholinesterase inhibitors such as donepezil, galantamine (razadyne), and rivastigmine, memantine, monoamine oxidase type B inhibitor (Selegiline), and antipsychotics such as olanzapine and risperidone (however, they are still not approved by the U.S. Food and Drug Administration for the treatment of Alzheimer's disease) [38]–[40].

AChEIs are considered the first-line agents for the treatment for mild to moderate AD, as they enhance cholinergic neurotransmission. The latest guidelines recommend that these medicines should be continued in the later (severe) stages of the disease. However, a Cochrane review concluded that treatment with AChEIs for six months to one year improved cognitive function by an average of −2.7 points on the ADAS-cog [38].

On the other hand, memantine functions by blocking the effects of glutamate. Memantine is usually prescribed in combination with AChEIs to reduce the doses in patients who cannot tolerate its side effects. Nevertheless, the effectiveness of memantine in patients with moderate to severe AD remains uncertain. A Cochrane review suggested that memantine at a dosage of 20 mg per day over six months can improve cognition (3 points on the SIB- Severe Impairment Battery measurement) and the ability to perform activities of daily living (1.3 points on the ADCS-ADL) in patients with moderate to severe AD [38],[39]. Another meta-analysis study, that included three trials with 431 patients with mild AD and 697 patients with moderate AD, found no significant differences between memantine and the placebos for patients with mild AD [41].

Although the combined use of donepezil and memantine significantly reduced the average number of hospital or emergency department visits per year by 0.078 (13.8%), 0.144 (25.5%), and 0.132 days (23.4%), respectively [42], a recent meta-analysis study was conducted to quantitatively assess the efficacy and safety of AChEIs, memantine, and sodium oligomannate (GV-971) in AD treatments. This study analyzed clinical trials characterized by randomization, placebo-control, and double-blinded methodologies using the Review Manager Version 5.4 software and Molecular docking in the result analysis. The study found that all drugs improved cognitive function; however, on the Neuropsychiatric Inventory measurement (NPI), only 10 mg of donepezil and 24 mg of galantamine showed improvements. On ADCS/ADL, only 20 mg memantine and 900 mg GV-971 had no notable distinction from the placebo. In addition, donepezil 5 mg and GV-971 900 mg did not raise the drug withdrawal rates [43].

Another study aimed to compare AChEIs with memantine, and performed a double-blind, placebo-controlled trial with random assignments to either the cholinesterase inhibitor or memantine. The results showed that behavioral benefits were only observed for the 10 mg daily donepezil group (−2.72, 95% CI −4.92 to −0.52) and for the 24 mg daily galantamine trial (−1.72, 95% CI −3.12 to −0.33). In contrast, only the 5 mg daily donepezil trial had no outcomes. Moreover, compared to the placebo, dropouts and adverse events observed with cholinesterase inhibitors but not with memantine [44].

All these studies indicate that current medications for AD do not prevent disease progression but only delay the deterioration symptoms, with varying based on disease stage and targeted symptoms.

### 2.6. Novel approaches to AD treatment

Over the past two years, research and experiments have focused on a new therapeutic perspective: targeting molecular metabolic approaches based on modern hypotheses of AD's mechanism. This paragraph highlights the latest experiments published on PubMed that explored innovative treatment approaches, specifically targeting calcium channels, the alpha-7 nicotinic acetylcholine receptor, and adiponectin. Moreover, it reinforces the established theories in this field, including the significant correlation between AD and type 2 diabetes, as well as the potential role of malaria drugs (dihydroartemisinin-piperaquine) in enhancing neuroplasticity.

One in-vivo study aimed to explore the biochemical regulation of the interaction between the hippocampal pathway member WWC1 (a key component of AMPA-type glutamate receptors, crucial for synaptic plasticity) along with the kinases LATS1 and LATS2 (LATS1/2) in primary hippocampal neurons. The results showed that the pharmacological inhibition of MST1/2 (mammalian Sterile 20-like kinase 1/2) in organoids promoted the dissociation of WWC1 from LATS1/2, thus leading to an increase in WWC1 within AMPAR-containing complexes. This potentially enhanced the synaptic transmission and improved cognition in healthy male mice and male mouse models of AD and ageing [45].

Calcineurin, a calcium and calmodulin-dependent serine/threonine protein phosphatase, plays crucial roles such as regulating the transcription factor NF-AT during T-cell activation, mediating microbial responses, and serving as part of a critical signaling pathway for learning and memory [46],[47]. However, neuronal calcineurin may be hyperactivated in AD. Hence, a study conducted in a 3  ×  Tg-AD transgenic mouse model of AD, published by Zeng et al., investigated the effects of FK506 (a calcineurin inhibitor) on Alzheimer-like behavior and synaptic dysfunction. The results showed that FK506 treatment reduced cognitive impairments, lowered the levels of specific characteristics of postsynaptic deficits, including PSD-95 and NR2B, and reversed the long-term potentiation deficiency and dendritic spine impairments [47].

Furthermore, the metabolic molecular interaction between calcium signals, N-methyl-D-aspartate receptors (NMDARs), which are glutamatereceptors, L-type voltage-gated calcium ion channels (L-VGCCs), ryanodine receptors (RyRs), and store-operated calcium entry (SOCE) are crucial in neuronal activity and synaptic plasticity, as well as to regulate gene expression, transcription, and alternative splicing [48]–[50].

In many neurodegenerative diseases, including AD, calcium homeostasis dysregulation is a commonly observed cellular defect [50]. One lab study was conducted using primary cortical neurons (DIV15) derived from C57BL/6 WT and APPswe/PS1dE9 (APP/PS1) double-transgenic AD mice. Neurons were exposed to APOE4 and observed. The findings showed that exposed neurons exhibited the suppression of both basal and NMDAR-mediated protein synthesis responses, which was attributed to the disruption of calcium homeostasis (then, the results were validated in a human stem cell-derived neuron model of AD). These findings not only suggest promising future treatments but also highlight that alterations in calcium signaling may serve as an early marker of neurodegeneration and, consequently, an early diagnostic indicator for AD [50].

The entorhinal cortex plays a crucial role in forming new memories, partly due to neuropeptide cholecystokinin (CCK), which is released from the entorhinal cortex and facilitates neocortical-associated memory and long-term potentiation [51]. Hence, the researchers examined mRNA expressions of CCK and the CCK-B receptor (CCKBR) in two mouse models, 3 × Tg AD and CCK knock-out (CCK−/−) mice, and measured cognition function with the Morris water maze and neuroplasticity with in-vitro electrophysiological recording. Subsequently, drugs were given to mice to investigate the rescue effects on cognitive loss or directly applied to brain slices to explore the influence on long-term potentiation. The results demonstrated that aged 3 × Tg AD mice exhibited reduced CCK mRNA expression in the entorhinal cortex, decreased CCKBR expression in the neocortex and hippocampus, and impaired cognition and neuroplasticity comparable with CCK−/− mice. Nevertheless, the animals exhibited an improved performance and enhanced long-term potentiation after the treatment of CCKBR agonists. These findings may provide new light on the future pharmaceutical directions for AD and dementia [51].

The alpha7 nicotinic acetylcholine receptor (α7 nAChR) is another key component of the cholinergic nerve system in the brain, playing a crucial role in excitatory neurotransmission, modulating the release of various neurotransmitters, regulating neurite outgrowth, activating molecular pathways during neurogenesis, and supporting cognitive functions. Moreover, α7 nAChR dysfunction or dysregulated expression could be found in neurodegenerative diseases such as AD [52]. Therefore, several studies were conducted to investigate the potential interventions on these receptors. A recent laboratory study treated APP/PS1 and wild-type (WT) mice with selective agonist PNU (or saline) for 7 days at the ages of 6 and 10 months, followed by a Morris water maze analysis to investigate the mechanism underlying the effect of α7 nAChR activation. The results revealed that the activation of α7 nAChR by PNU improved the learning and memory of mice carrying the APP/PS1 mutation, regulated enzyme levels involved in APP metabolism to reduce β-amyloid peptide damage, decreased oxidative stress, and sustained synaptic plasticity, possibly through the enhancement of the nuclear factor erythroid 2-related factor (Nrf2) transcription factor/Hemoxygenase 1 (HO-1) (Nrf2/HO-1) pathway that activates the expression of various neuroprotective genes that code for anti-oxidant, anti-inflammatory, and detoxifying proteins [53]–[55].

Microglia-neuron interactions are an essential component of the brain's immune system and play a crucial role in synapse remodeling, the inflammatory response, and antigen presentation, all of which are part of the AD epidemiology [56]. However, in aged brains, microglia become hypersensitive to neurotoxic and inflammatory insults, thereby expressing high levels of pro-inflammatory mediators and reactive species while exhibiting deficits in phagocytosis and motility [56]. Hence, one study investigated the effects of Cordycepin treatment (CCs) in 9-month-old APP/PS1 mice and monitored the potential modifications through behavioral tests [57]. Cordycepin is well known for its anti-cancer, anti-oxidant, and anti-inflammatory properties. This substance, produced by the entomopathogenic fungus Cordyceps, interferes with various biochemical and molecular processes, including purine biosynthesis, DNA/RNA synthesis, and mammalian target of rapamycin (mTOR) signaling transduction [58]. The findings indicated that CCS polarized the microglia from M1 to the M2 phenotype, thereby activating the cAMP-response element-binding protein (CREB), inhibiting neuronal apoptosis, and promoted synaptic remodeling. This was accompanied by the in vivo and in vitro upregulation of NGF. Furthermore, the sg3 promoter region of NGF (−1018 to −1011) was identified as the key binding site for CREB-induced NGF transcription, thus enhancing NGF expression and secretion. These results offer a novel perspective on anti-AD therapy [57].

The endocrinologic aspect of neuroplasticity should also be considered, particularly the role of the adipocyte-secreted hormone adiponectin. Adiponectin is a cytokine that functions as a metabolic regulator capable of crossing the BBB, modulating neuronal activity in various brain regions, and improving neuronal metabolism in different animal models, including those of obesity, diabetes, and AD [59].

A recent study employed the Barnes maze test, the Morris water maze test, and fear conditioning test to evaluate the memory-ameliorating effects of adiponectin on 3 × Tg-AD mice. To assess changes in basic synaptic transmission, long-term potentiation (LTP), and long-term depression (LTD), in vivo hippocampal electrophysiological recording were conducted. Additionally, immunohistochemistry staining and western blot analyses were used to evaluate microglia and astroglia activation, as well as the expression levels of proinflammatory factors and anti-inflammatory factor, including IL-10v. The results found that adiponectin ameliorated the cognitive deficits in 3 × Tg-AD mice by enhancing in vivo synaptic plasticity and reducing neuroinflammation in the hippocampus [60].

These findings can be explained through the metabolic regulation of synaptic plasticity. The continuous influx of peripheral glucose across the BBB is essential to maintain the central glucose levels [59]. Insulin receptors play a crucial role in regulating glucose entry into neurons. In aged adiponectin knockout mice, the chronic absence of adiponectin leads to insulin resistance in the hippocampus, characterized by the decreased phosphorylation of AMPK, Akt, and PI3K, despite normal insulin plasma levels and insulin receptor expression in the hippocampus. However, impairment in Akt phosphorylation in response to intra-hippocampal insulin administration was reversed by adiponectin in cultured insulin-resistant neuronal cells [59].

Recent studies have explored the potential correlation between AD and type 2 diabetes mellitus (T2DM). Although researchers found similarities between the two diseases based on gene ontology (GO) and protein-protein interactions (PPIs), for instance, the hub gene SLC2A2 (which encodes the transmembrane carrier protein GLUT2) connects the most DEGs in both AD and T2DM [61],[62], no definitive evidence has yet established a causal link. However, a study published in 2023 investigated whether the novel glucagon-like peptide-1 (GLP-1)/glucose-dependent insulinotropic polypeptide (GIP) receptor agonist DA4-JC provided protective effects in the triple APP/PS1/tau mouse model of AD using in vivo LTP recordings of the hippocampus, biochemical analyses of biomarkers, and quantified synapses using the Golgi method. The results found that DA4-JC improved cognitive impairment (assessed through a reversal MWM task). It enhanced LTP in the hippocampus, upregulated post-synaptic density protein 95 (PSD95) and synaptophysin (SYP), improved mitochondrial function, and activated the phosphatase and tensin homologue induced putative kinase 1 (PINK1)-Parkin mitophagy signaling pathway while downregulating amyloid, p-tau, and the autophagy marker P62. These findings suggest that anti-T2DM drugs could serve as a promising treatment for AD and further substantiate the link between the two diseases [63].

Histone H3 lysine 9 di-methylation (H3K9me2) and histone H3 lysine 9 tri-methylation are epigenetic markers associated with the silencing of heterochromatin domains at repetitive DNA elements [64]. H3K9me2 contributes to aberrant DNA repair, regulates cell type-specific gene expression, and affects non-histone targets such as transcription factors, steroid hormone receptors, and histone-modifying enzymes [65]. However, increased H3K9me2 levels have been observed in the prefrontal cortex of AD patients, particularly at the promoter region of neprilysin (NEP) gene, which encodes a protein responsible for of Aβ peptide degradation. Additionally, elevated expression levels of EHMT1 (which catalyzes the dimethylation of H3K9) were detected, while the AMPA and NMDA glutamate receptor subunits were decreased [64].

Nevertheless, increased H3K9me2 levels in the hippocampus are also catalyzed by lysine methyltransferase G9a, which forms a heteromeric complex with the G9a-like protein (GLP). Since G9a/GLP is involved in regulating synaptic plasticity, researchers have suggested that inhibiting G9a/GLP receptors could improve cognitive functions in AD patients [66]. Han tested whether G9a/GLP inhibition could restore LTP deficits in the CA1 region of hippocampal slices from APP/PS1 mice. A long-lasting, input-specific potentiation was observed after the perfusion of APP/PS1 hippocampal slices with either the G9a/GLP inhibitor BIX-01294 (BIX, 500 nM) or UNC-0642 (UNC, 150 nM) during LTP induction using a strong tetanization protocol (STET). The results showed that a G9a/GLP blockade during LTP induction could rescue input-specific synaptic potentiation in APP/PS1 hippocampal slices through upregulating protein synthesis. However, when researchers tested whether prior applications of G9a/GLP inhibitors alone could convert the weak tetanization protocol (WTET)-induced early-LTP into late-LTP, only a transient potentiation to input S2 after WTET. These findings suggest that acute G9a/GLP inhibition alone cannot induce cell-wide priming and the synthesis of plasticity-related products (PRPs) under AD-like condition, despite prior studies reporting the upregulation of synaptic genes and synaptic transmission in Aβ-impaired cortical neurons and familial AD (FAD) mouse models in vivo. The researchers in this study attributed these results to either a lack of priming by G9a/GLP inhibition or the duration of G9a/GLP inhibitor treatment [66]. Although these results are interesting, further clinical studies are needed to validate the effectiveness of G9a/GLP inhibitors as a potential treatment for AD disease.

Some researchers suggested that neural plasticity could be enhanced using well-known malaria drugs. Dihydroartemisinin–piperaquine (DHA–PPQ), an active metabolite of sesquiterpene trioxane lactone, has been a first-line treatment for uncomplicated falciparum malaria in Indonesia since 2010 [67],[68]. DHA–PPQ specifically activates the autophagy-lysosomal degradation system, which removes harmful substances and maintains cell viability, a process crucial for neuroprotectivity in the CNS, thus potentially preventing neurodegenerative illnesses such as AD. Only a few in vivo studies have investigated this effect further. One recent study constructed an adeno-associated virus carrying hTau cDNA (AAVhTau) and then microinjected it through the intra-hippocampal region to establish a mouse model of tauopathy. Through a combination of behavioral tests, electrophysiological recordings, and western blotting assays, the results found that DHA increased the hippocampal CA1 LTP, induced protein O-GlcNAcylation modification, and reduced protein phosphorylation. Establishing crosstalk between O-GlcNAcylation and phosphorylation suggests DHA as a potential therapeutic agent to improve learning and memory deficits associated with the tau pathology [69].

Recent studies have explored the potential of cannabinoids in managing neuropsychiatric symptoms associated with AD. A randomized placebo-controlled trial of Nabilone, a synthetic cannabinoid, demonstrated its effectiveness in reducing agitation in AD patients, thus offering a promising therapeutic option [70]. Additionally, a study on tetrahydrocannabinol (THC) revealed its potential in alleviating neuropsychiatric symptoms in dementia patients, further supporting the role of cannabinoids in dementia management [71]. Beyond THC and Nabilone, minor phytocannabinoids have also emerged as promising candidates for neuroprotection, with recent systematic reviews highlighting their neuroprotective potential in various neurological disorders [72]. Furthermore, novel therapeutic strategies, such as psychedelic treatments, are being investigated for their potential to address AD comorbidities, thus opening new avenues for research in neurodegenerative disease management [73].

## 3. Conclusions

In this review, we presented the latest studies on the pathological mechanisms underlying AD, thereby highlighting multiple contributing factors, including genetic mutations, the gut-brain axis, neuroinflammation, sex hormones, and synaptic collapse. These mechanisms collectively contribute to the decline of neuroplasticity and the onset of cognitive symptoms. However, despite significant progress, there remains insufficient scientific evidence to pinpoint the exact underlying mechanisms of AD. Furthermore, we discussed the limitations of current treatment guidelines that primarily rely on AChEIs, which have shown limited efficacy in altering the disease progression. While several experimental therapies targeting neuroplasticity have demonstrated promise in preclinical studies, their clinical translation remains a challenge. Given the growing body of research on alternative therapeutic approaches, including nootropics, plastogens, and natural products, the future treatment guidelines should integrate these emerging strategies where evidence supports their efficacy. Based on the findings of this review, we encourage further research into neuroplasticity-targeting treatments while also advocating for an expanded, evidence-based approach that incorporates complementary and alternative therapies. Physicians and researchers should consider these insights when designing more effective, personalized interventions for AD patients.

## Use of AI tools declaration

The authors declare they have not used Artificial Intelligence (AI) tools in the creation of this article.
